# Gold nanorod reshaping *in vitro* and *in vivo* using a continuous wave laser

**DOI:** 10.1371/journal.pone.0185990

**Published:** 2017-10-18

**Authors:** David Harris-Birtill, Mohan Singh, Yu Zhou, Anant Shah, Pakatip Ruenraroengsak, Maria Elena Gallina, George B. Hanna, Anthony E. G. Cass, Alexandra E. Porter, Jeffrey Bamber, Daniel S. Elson

**Affiliations:** 1 Hamlyn Centre for Robotic Surgery, Imperial College London, London, United Kingdom; 2 Department of Surgery and Cancer, Imperial College London, London, United Kingdom; 3 Department of Chemistry, Imperial College London, London, United Kingdom; 4 Joint Department of Physics and CRUK Cancer Imaging Centre, Institute of Cancer Research and Royal Marsden NHS Foundation Trust, Sutton, London, United Kingdom; 5 Department of Materials and London Centre for Nanotechnology, Imperial College London, London, United Kingdom; Institute of Materials Science, GERMANY

## Abstract

Gold nanorods (GNRs) are increasingly being investigated for cancer theranostics as they possess features which lend themselves in equal measures as contrast agents and catalysts for photothermal therapy. Their optical absorption spectral peak wavelength is determined by their size and shape. Photothermal therapy using GNRs is typically established using near infrared light as this allows sufficient penetration into the tumour matrix. Continuous wave (CW) lasers are the most commonly applied source of near infrared irradiation on GNRs for tumour photothermal therapy. It is perceived that large tumours may require fractionated or prolonged irradiation. However the true efficacy of repeated or protracted CW irradiation on tumour sites using the original sample of GNRs remains unclear. In this study spectroscopy and transmission electron microscopy are used to demonstrate that GNRs reshape both *in vitro* and *in vivo* after CW irradiation, which reduces their absorption efficiency. These changes were sustained throughout and beyond the initial period of irradiation, resulting from a spectral blue-shift and a considerable diminution in the absorption peak of GNRs. Solid subcutaneous tumours in immunodeficient BALB/c mice were subjected to GNRs and analysed with electron microscopy pre- and post-CW laser irradiation. This phenomenon of thermally induced GNR reshaping can occur at relatively low bulk temperatures, well below the bulk melting point of gold. Photoacoustic monitoring of GNR reshaping is also evaluated as a potential clinical aid to determine GNR absorption and reshaping during photothermal therapy. Aggregation of particles was coincidentally observed following CW irradiation, which would further diminish the subsequent optical absorption capacity of irradiated GNRs. It is thus established that sequential or prolonged applications of CW laser will not confer any additional photothermal effect on tumours due to significant attenuations in the peak optical absorption properties of GNRs following primary laser irradiation.

## Introduction

Noble metal nanoparticles have unique optical properties, predominantly determined by their size and shape. These properties may be utilised in biomedicine, as they may be applied systemically to provide image contrast, photothermal therapy and drug delivery for cancer diagnosis and treatment [1–4]. Gold nanorods (GNRs) are particularly useful in photothermal therapy of cancer [2,4,5–10] as their longitudinal surface plasmon resonance (LSPR) absorption peak can be tuned to absorb light very strongly in the visible or near infrared (NIR) region where tissues are relatively transparent. Photothermal therapy using GNRs is a useful treatment modality in that it can be targeted to work specifically at the site of cancer, be applied in a minimally invasive manner when coupled with endoscopic delivery, and be administered rapidly for theranostic (diagnostic and therapeutic) procedures with the potential for further application. The site-specific and immediate photothermal effect from the irradiation of GNRs within a tumour could potentially permit prompt recovery, be associated with fewer side-effects and a much shorter hospital stay than other established treatment modalities such as surgery or chemo-radiation. When established with NIR light, a deep photothermal effect can be achieved due to lower optical attenuation by haemoglobin within tissues, when compared with the attenuation by visible light. Previous studies have shown that applying highly energetic pulsed nanosecond or femtosecond laser light pulses cause nanorods to deform or change shape *via* a melting process [11–19]. This shape change causes the nanorod optical absorption peak to shift, and the optical absorption may decrease.

Continuous wave (CW) lasers with fluences of 2–10 W/cm^2^ were used in several studies for testing photothermal therapy with GNRs [4–8]. As CW lasers are generally less expensive, smaller and more portable than their pulsed counterparts, they are currently the more clinically compatible of the two types of photothermal therapy sources. It has also been shown by Kirui *et al*. [20] that there may be potential for fractionating the irradiation regime and delivering CW laser light over several rounds of therapy, using a laser fluence of 5 W/cm^2^. It has also been shown by Mohamed *et al*. [21] that heating with only a thermostatically controlled water bath can alter the GNR shape, decreasing their aspect ratio and LSPR peak wavelength by increasing temperature. When tumours are large, photothermal therapy using GNRs may require repeated application. However it has not yet been established whether re-application of CW laser to a previously irradiated site would be as photothermally effective as the initial laser application in the presence of pre-existing irradiated GNRs within the tumour site. This is because CW laser-induced GNR shape change has not yet been investigated and is thus assessed by this study. This is a crucial factor since any reduction in the absorption coefficient at the therapeutic laser wavelength due to GNR reshaping will reduce the heating effect and thus reduce the impact of the laser thermal therapy for subsequent irradiation at the same wavelength, making it significantly less effective.

Remote monitoring of the thermal heating of GNRs can be achieved, in solution and in tissue, using a thermal imaging camera. For instance, Chou *et al*. [22] have shown a reduction in GNR sample heating after 5 and 60 seconds of 146 mW CW laser irradiation (785 nm), both when the sample was diluted and when the LSPR peak was at a different wavelength. However neither an analysis of the nanorods’ shapes nor their spectra post CW laser irradiation was presented in this paper. Although GNR laser heating is dependent on the optical absorption, thermal imaging does not provide a direct measure of optical absorption; therefore it cannot measure GNR bleaching. The most common approach to measure changes in GNR optical properties is using optical spectroscopy. However this is best done before and after irradiation, as during irradiation the laser light may saturate the photodetector (normally a CCD or photodiode) within the spectral region of interest, and can therefore not be used to monitor the shape change dynamics during therapy.

Photoacoustics (PA) uses the optical absorption of pulsed laser light by materials to generate an acoustic wave that may be detected by an ultrasound transducer [23]. GNRs have been shown to be a good PA contrast agent due to their strong optical absorption [24–29]. Therefore PA should be able to monitor the destruction and reshaping of GNRs over time due to laser heating by a high power CW laser.

## Materials and methods

### Gold nanorod synthesis

GNRs were produced using the seed-mediated approach described by Yang *et al*., using hexadecyltrimethylammonium bromide (CTAB) as the surfactant [30]. Due to the variability in the absorption peak wavelength when creating GNRs using this method, many batches were made with different concentrations of silver nitrate until the absorption peak of the GNRs was at the appropriate wavelength of approximately 808 nm (measured with Perkin Elmer Lambda 25 UV/Vis Spectrophotometer, Waltham USA), the emission wavelength of a particular NIR laser diode. The GNR solution was centrifuged at 8500 rotations per minute for twenty minutes, the supernatant decanted, and the GNRs were re-suspended in water and centrifuged with the same settings to remove excess CTAB. Three different batches of GNRs (samples ***α***, ***β*** and ***γ***) were tested to check repeatability *in vitro*. For *in vivo* experiments, multifunctionalised GNRs (PEG-GNR-Cy5.5-Anti-EGFR-antibody) were used in immunodeficient rodents.

### Irradiation and monitoring

A preliminary validation experiment on concentrated GNR solution samples contained within optically transparent cuvettes (GNRs with an optical density of 0.75 at 808 nm, which corresponded to the emission peak of our CW laser) was tested with CW laser irradiation and the general effect on the GNRs post irradiation was visualised with a transmission electron microscope (TEM), (TEM JEOL JEM-2000FX, Tokyo Japan). Following this preliminary experiment, the actual study group consisted of 3 ml aliquots of a newly prepared GNR solution (with varying optical densities) which were pipetted into an optically transparent membrane dish and irradiated for 5 minutes using a CW laser (DenLase-810/7, Beijing China) emitting at 808 nm with a power of 6 W and a beam area of 1 cm^2^ at the sample surface. The solution was then removed and the dish was washed twice with water. One aliquot of GNRs was also used to evaluate the effect of increased CW laser fluence. For this sample the beam area was reduced to 0.5 cm^2^ while maintaining 6 W laser power, effectively increasing the fluence to 12 W/cm^2^. For this case the irradiation time was reduced to 45 seconds, because the solution heated so rapidly that it started to boil.

**Fig 1:**
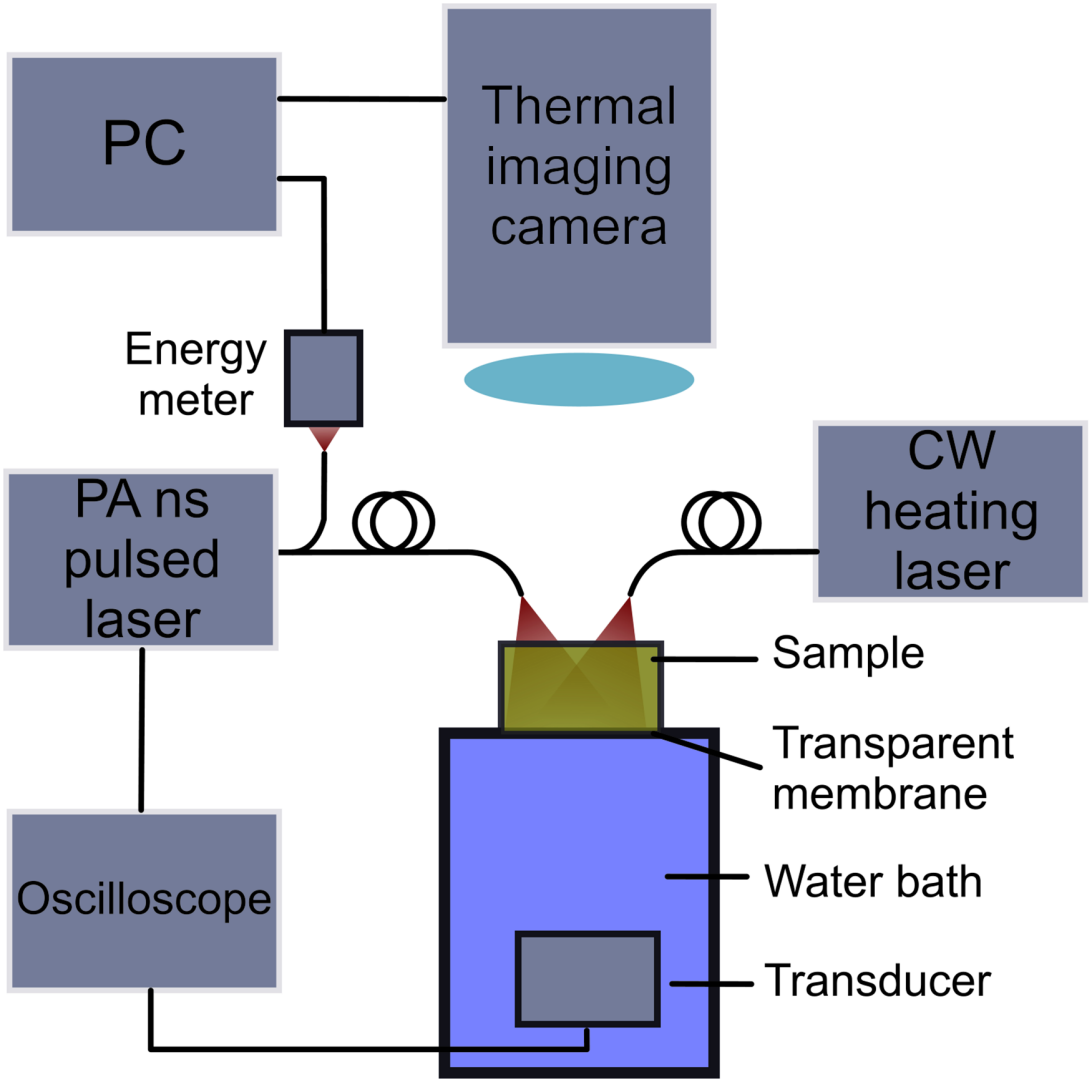
The experimental set-up for monitoring GNR reshaping during CW laser application, using both PA and thermal imaging. Curved lines represent optical fibres and straight lines represent electronic connections.

To monitor the heating and GNR destruction, thermal imaging and photoacoustics were used; the experimental set-up is shown in Fig 1. To enable acoustic transmission from the sample to the ultrasound transducer, the base of the sample dish was in contact with the water bath below, and any bubbles under the membrane were removed before starting. A very thin Mylar^™^ membrane was intentionally used (75 μm thick) to ensure minimal acoustic attenuation. For the PA light source, a Nd:YAG pumped optical parametric oscillator (OPO) laser (Quantel Brilliant B with Rainbow OPO, Les Ulis France) was used which had a 7 ns laser pulse at a 1 Hz repetition rate, a wavelength of 808 nm, a measured mean energy of 20.3 ± 1.5 mJ per pulse (where the error is the standard deviation measured over all 1800 pulses during the experiment) and with a beam area at the sample surface of 200 mm^2^. To check that the PA laser pulse did not cause GNR reshaping, a control was made for each sample with the CW laser switched off, and these samples were removed and analysed with spectroscopy and electron microscopy. The CW and PA lasers were at the LSPR wavelength of the GNRs. This enabled monitoring of any changes occurring to the GNRs arising from the CW laser using PA signal, as the GNRs would potentially be reshaped from the CW laser at that wavelength. This further enabled investigation into the reshaping effects arising from both lasers being delivered at the same wavelength.

For each laser pulse, a PC recorded the laser energy detected by the energy meter (Gentec-eo Solo2, Quebec, Canada). An oscilloscope recorded the PA pulse detected by the ultrasound transducer in response to the laser energy absorbed by the sample, using an electronic TTL (Transistor-Transistor Logic) pulse from the laser at the time of Q-switching as a trigger. The ultrasound transducer has a measured 6.5 MHz centre frequency with a bandwidth of 4.5 MHz full width at half maximum (FWHM) amplitude, with a distance to focal maximum of 73.5 mm and FWHM pressure amplitude beam width of 17.5 mm axial by 1 mm radial measured using a beam profiling system (Precision Acoustics, UMS). The thermal imaging camera (FLIR Systems ThermaCAM S65, Wilsonville, USA) was manually triggered at the beginning of the irradiation and set to acquire images at 1 Hz frame rate for the length of the irradiation. The thermal imaging camera has a field of view of 24° x 18°, with a thermal sensitivity of 0.08°C, a detector resolution of 320x240, a spectral range of 7.5 to 13 μm and a reading accuracy of ± 2%. The CW laser was also manually triggered at the start of the PA and thermal imaging acquisitions.

### Chemical and TEM analysis of *in vitro* samples

Samples from before and after CW irradiation were placed into cuvettes and the absorbance spectra were measured using the transmission spectrophotometer. The maximum absorbance value for the pre-irradiated spectra for each sample type was measured. During subsequent analysis, all GNR samples were normalised to the same pre-irradiation maximal absorbance intensity by dividing the pre-laser optical absorbance spectra by the maximum absorbance value pre-irradiation. A drop of each sample was then placed onto a carbon grid and air dried for imaging with TEM at 25,000x magnification. This allowed visualisation of the shape changes that occurred. The length and width of each distinguishable GNR in all the images acquired for all samples were also measured manually by locating the ends of each GNR in the images, and ignoring the spheres present, using ImageJ to measure the lengths, and then locating the centre to measure their widths. The aspect ratio of each GNR was calculated by dividing the length by the width, and the mean and standard deviation of the lengths, widths, and aspect ratios were then calculated.

### Thermal image analysis

For each acquisition a region of interest (ROI) was selected for the sample and for a background region. The mean and standard deviation of the pixels within the ROI for each frame were then calculated. Any change in background temperature was subtracted from the sample temperature to provide a background corrected temperature rise over time due to laser heating. During analysis, the temperature measurements were normalised to the relative concentration of each GNR sample.

### PA data and pulsed laser energy

**Fig 2:**
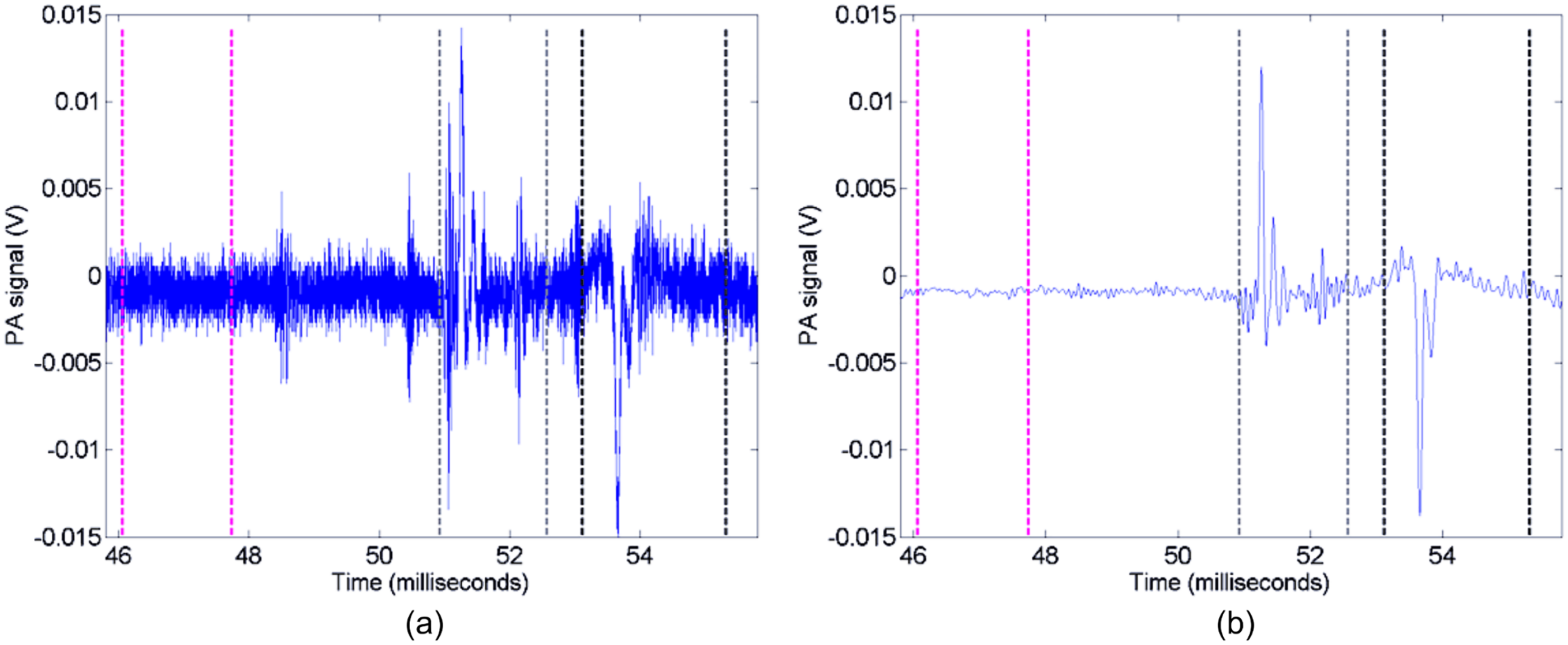
Example measured PA response (a), and after low pass filtering (b). Here the time periods corresponding to the background (magenta), the bottom of the sample (grey), and the top of the sample (black) are indicated between the vertical dashed lines.

A low pass filter (cut-off at 15 MHz) was applied in the frequency domain using Matlab to remove the PA high frequency noise, setting all frequency content above 15 MHz to zero, as illustrated in Fig 2. Three intervals of the temporal PA response were extracted for analysis: one corresponding to the PA response from the top of the sample (*V*_*top*_), one from the bottom of the sample (*V*_*bottom*_), and one for the background (*V*_*background*_), as illustrated by the dotted vertical lines in Fig 2. The absolute value of the PA voltage response for each laser pulse was integrated over these intervals and divided by the interval time to obtain a measure of the sample PA strength (derived from *V*_*top*_ and *V*_*bottom*_), and the background level (derived from *V*_*background*_). The final PA signal was calculated by subtracting the background level from the sample PA strength and was normalised by the corresponding laser energy for each pulse (*F*(*t*)) using this equation:

$$V_{sig}\left(t\right)=\frac{V_{top}\left(t\right)+V_{bottom}\left(t\right)-2V_{background}\left(t\right)}{F\left(t\right)}$$ (1)

This was repeated for each pulse in the sequence and for every sample.

### *In vivo* analysis of GNRs in tumour-bearing and tumour-irradiated mice

This study was carried out in strict accordance with the Animals (Scientific Procedures) Act 1986. The protocol was approved by the Home Office and Secretary of State (UK) (project licence number PPL 70/7996). All efforts were made to minimise any suffering. Immunodeficient BALB/c nu/nu mice were inoculated subcutaneously with a human oesophageal adenocarcinoma cell line (FLO-1 cells) and developed solid tumours of at least 5 mm prior to any intervention. FLO-1 cells were established from a primary distal oesophageal adenocarcinoma in a 68 year-old white male in 1991. They are of epithelial origin and have an adherent growth mode. Study mice were then injected intratumourally with 50 μl of aqueous GNR solution. These GNRs were PEGylated and functionalised with a fluorophore (Cy 5.5) modified with anti-EGFR antibody, and had an OD_λ-808nm_ of 13.2. In one group of three mice, tumour sites were biopsied for microscopic examination for the presence of intracellular GNRs. In another group of three mice, tumours that received intratumoural (IT) injections of GNRs were then irradiated with the same CW NIR laser used in the previous *in vitro* study but now emitting at a power of 1.6 W with a beam diameter of 6 mm at the tumour surface for three minutes. A control group of three mice with tumours was evaluated where NIR irradiation was commenced for three minutes without any GNRs being administered. This was to assess and quantify the mean temperature rise and photothermal effect, if any, seen arising independently from the CW laser alone. NIR irradiation was performed under general anaesthesia (with an intraperitoneal injection of ketamine and xylazine) alongside post-procedural analgesia on the same day of administering GNRs. Temperature changes occurring within the irradiated tumour site was measured with the thermal imaging camera mounted above the mouse. Temperature rise analysis was performed in a manner akin to the *in vitro* study, with subtraction from background and baseline temperatures. In total 9 mice were used in this study.

**Fig 3:**
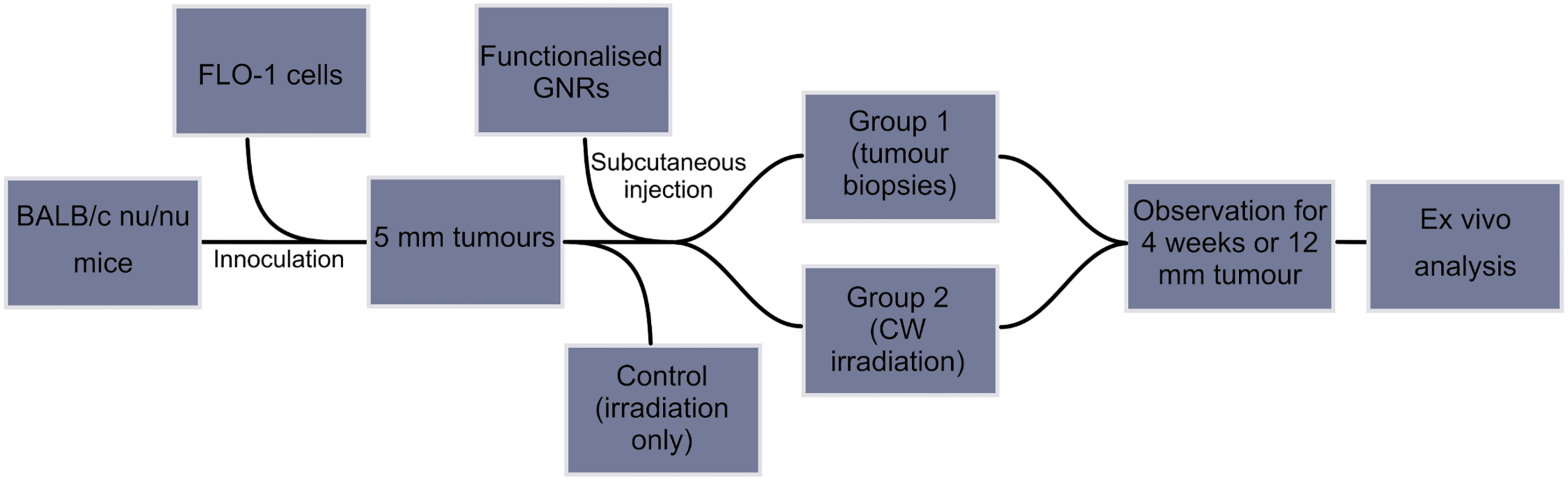
Scheme of experimental procedure of *in vivo* analysis.

Tumours sites and the animals were examined on the day of GNR administration and daily until day 30 post-irradiation. One half of the tumour sample was processed for microscopic evaluation of tumour cells, while the other half of the sample was analysed using bright field transmission electron microscopy (TEM JEM 2100F) and energy-dispersive X-ray spectroscopy (EDX, X-Max 80 mm^2^, Oxford Instruments, UK). The same examination was performed of irradiated tumour sites which were excised at day 30 post irradiation. The end point of the study was four weeks after the application of NIR light or when tumours exceeded 12 mm in any two axes. This involved humane termination of the mouse under National Cancer Research Institute guidelines using a Schedule 1 method under terminal general anaesthesia. No animal perished prior to the end point of the study. A scheme for the experimental procedure is illustrated in Fig 3.

### Transmission electron microscopy (TEM) analysis of *in vivo* tissues

Briefly, dissected tissue specimens were washed (x3) with 0.9% saline solution before being fixed with 2.5% glutaraldehyde in sodium cacodylate buffer solution (pH 7.4) for two hours at 4°C. The tissue specimens were then washed thrice with sodium cacodylate buffer solution, and stained with 1% osmium containing 2% potassium ferrocyanide for two hours, and washed with distilled deionised water (x3) and incubated overnight with 1% uranyl acetate in cacodylate buffer at 4°C before *en bloc* Walton’s lead aspartate staining was performed. The samples were subsequently dehydrated with 25%, 50%, 70%, 90% and 100% anhydrous ethanol for 15 minutes (x3) each, before being placed in anhydrous ice-cold acetone and left at room temperature for 20 minutes (x3). Durcupan ACM was used as the resin. The samples were then placed into 25%, 50% and finally 75% Durcupan: acetone for two hours each. Tissues were placed in 100% Durcupan overnight then into fresh 100% Durcupan for two hours. The resulting resin block was then sectioned with a diamond knife ultramicrotome. Each section was 50–70 nm thick, and placed on copper grids before viewing using TEM (TEM JEM 2100F, operated at 200kv) and EDX was used for further characterisation. At least 20 cells per ROI were observed from each tissue (n = 2) with total view of 40 cells.

## Results

This study demonstrates the consequences of using CW laser on the morphology and efficacy of GNRs, which is illustrated using results from spectroscopy and TEM from *in vitro* and *in vivo* settings. The notion that sequential or prolonged CW irradiation of tumour sites harbouring the same original GNRs would eventually lead to synergistic or summative effects on tumour regression akin to fractionated radiotherapy regimes on human cancers is tested here by *in vivo* experiments in mice with tumours. The results from our experiments would be useful in planning treatment for large tumours which will perceivably require multiple sessions of photothermal therapy.

**Fig 4:**
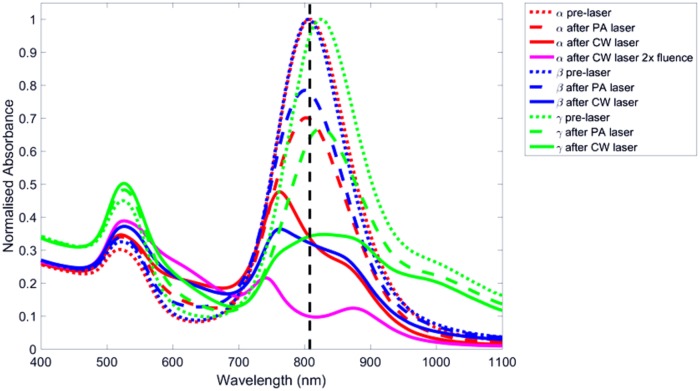
Measured absorbance spectra of original GNRs (dotted lines), after PA pulsed laser only (dashed lines), and after 6 W/cm^2^ CW laser light for 5 minutes (solid lines) across the three batches [red (sample *α*), blue (sample *β*) and green (sample *γ*)]. The magenta line refers to sample *α* after 12 W/cm^2^ laser light irradiation for 45 seconds. The black dashed line indicates the wavelength of the CW laser.

The absorbance of the samples showed consistent decreases in absorption of the LSPRs at the laser wavelength (the second peak) after irradiation of all three samples, as shown in Fig 4. With CW irradiation the spectral peak of GNRs shifted towards the blue, suggesting that the GNRs decreased in length (this is confirmed with their aspect ratio measurements in Table 1), while the absorption at wavelengths further from the laser was better preserved, suggesting the rods of the shape and size corresponding to high absorption at the laser wavelength are preferentially destroyed. There was a decrease in the GNR absorbance spectral peaks when irradiated with the PA pulsed laser, and yet the LSPR peak wavelength did not change, therefore these GNRs are unlikely to have changed to shorter rods as this would lead to a LSPR peak of shorter wavelength. This decrease in the longitudinal mode suggests a reduction in the number of GNRs, not a change in GNR length, as the spectral shape remains the same with PA pulsed wave laser irradiation. With CW laser the transverse mode peak (at around 530 nm) increased while their LSPR peaks not only diminished but their wavelengths also decreased, suggesting some GNRs may be converted into spheres, as spheres contribute to this transverse mode peak and blue shift in LSPR. The sample that received twice the CW laser fluence exhibited a further decrease in peak absorption and decrease in LSPR wavelength.

**Table 1:** The mean lengths, widths and aspect ratios of GNRs as measured manually from TEM images for two of the samples for each irradiation type. All errors show the standard deviation.

	Sample α pre-laser	Sample α after pulsed laser	Sample α after CW laser	Sample α after 2x fluence CW laser	Sample β pre-laser	Sample β after CW laser
Number of GNPs measured	203	122	566	485	140	87
Length (nm)	43.1 ± 7.7	42.1 ± 6.8	39.5 ± 7.5	35.8 ± 6.6	44.3 ± 4.9	34.8 ± 8.2
Width (nm)	11.5 ± 2.3	13.3 ± 2.2	14.4 ± 2.5	16.2 ± 2.6	11.6 ± 1.2	13.4 ± 2.8
Aspect ratio	3.8 ± 0.5	3.3 ± 0.8	2.9 ± 0.9	2.3 ± 0.8	3.9 ± 0.6	2.8 ± 1.0

Using the Beer-Lambert equation Absorption A = ε l c, where ε is the molar extinction coefficient 5.6 ± 0.4 x 10^9^ M^-1^ cm^-1^, l is the optical path length of 1 cm, the concentration of GNRs at the SPR_λ = 808nm_ in sample α was calculated to be 3.5 x 10^−8^ M, sample β was 2.7 x 10^−8^ M and sample γ was 1.7 x 10^−8^ M.

**Fig 5:**
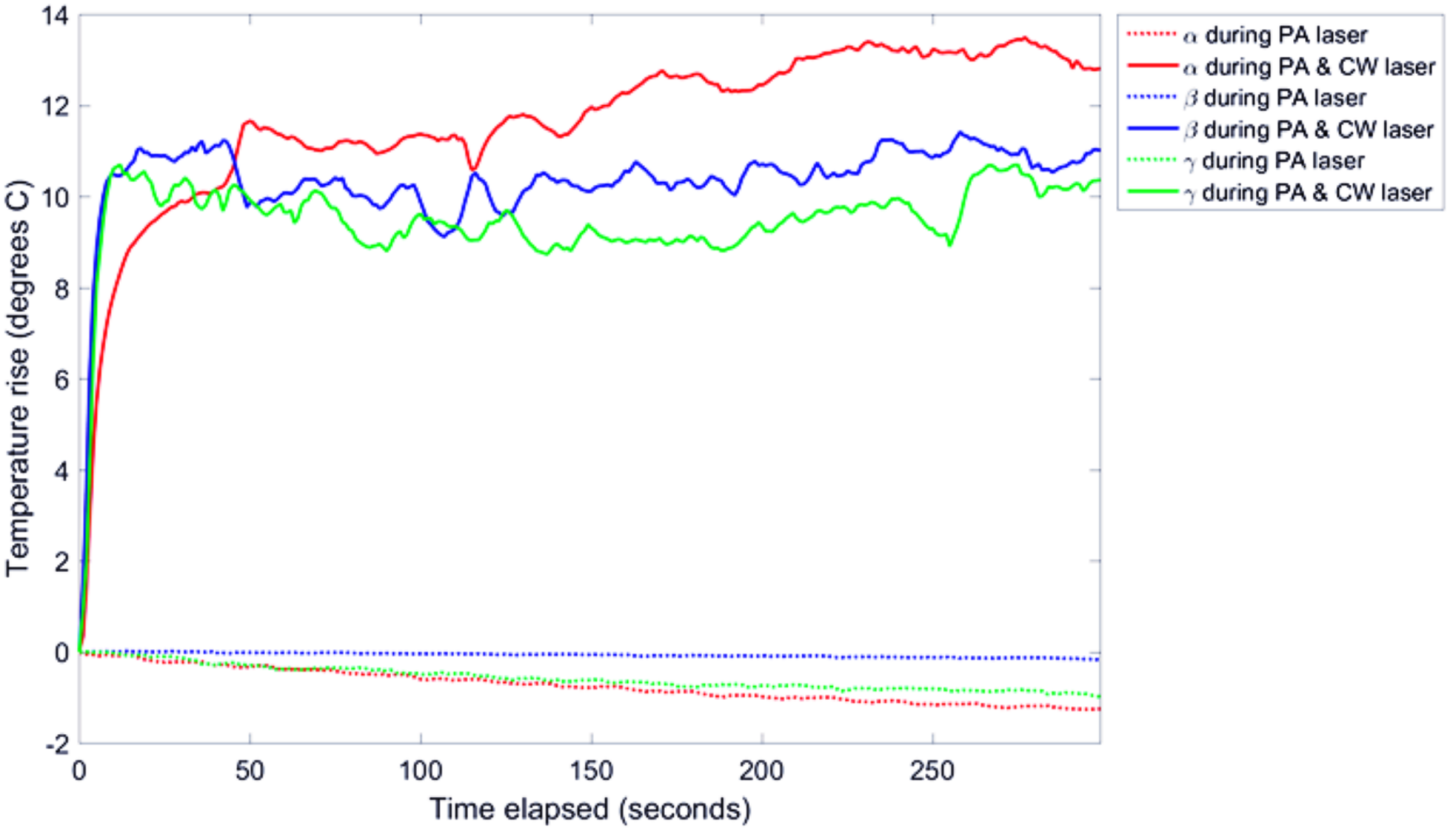
The measured temperature profiles from each sample with (solid lines) and without (dotted lines) CW laser irradiation using the same line colours used in Fig 1 to represent the different samples. GNR concentrations are labelled for each sample.

Sample *γ*, with results represented with a green line in Fig 4, had a slightly longer LSPR peak than the laser wavelength. Therefore rods with an aspect ratio corresponding to the LSPR peak were not heated optimally. Furthermore this sample was slightly less concentrated than the other samples, as shown by the original lower absorbance; both factors contributed to the lower temperature rise when compared with the other samples (as shown in Fig 5).

After measuring the lengths and widths, and subsequently calculating the aspect ratios of 1603 GNRs from TEM datasets, the mean dimensions were calculated for two of the three samples for all irradiation types, including before and after pulsed and CW laser irradiation, and these values are shown in Table 1. This consistently shows that the length of the rods is reduced while the width increased leading to a decreased aspect ratio. These length and width measurements also illustrate that nanorods reshape to become more spherical, which in turn explains the increase in the 530 nm transverse plasmon resonance peak seen in the absorption spectra of the irradiated samples in Fig 4. When twice the laser fluence was applied, a correspondingly larger effect was noticed.

The temperature profile from each sample, with and without the 6 W/cm^2^ CW irradiation is shown in Fig 5. All three samples heated dramatically during irradiation with a rise between 9 and 13.7°C, with the majority of the heating occurring within the first 10 seconds.

**Fig 6:**
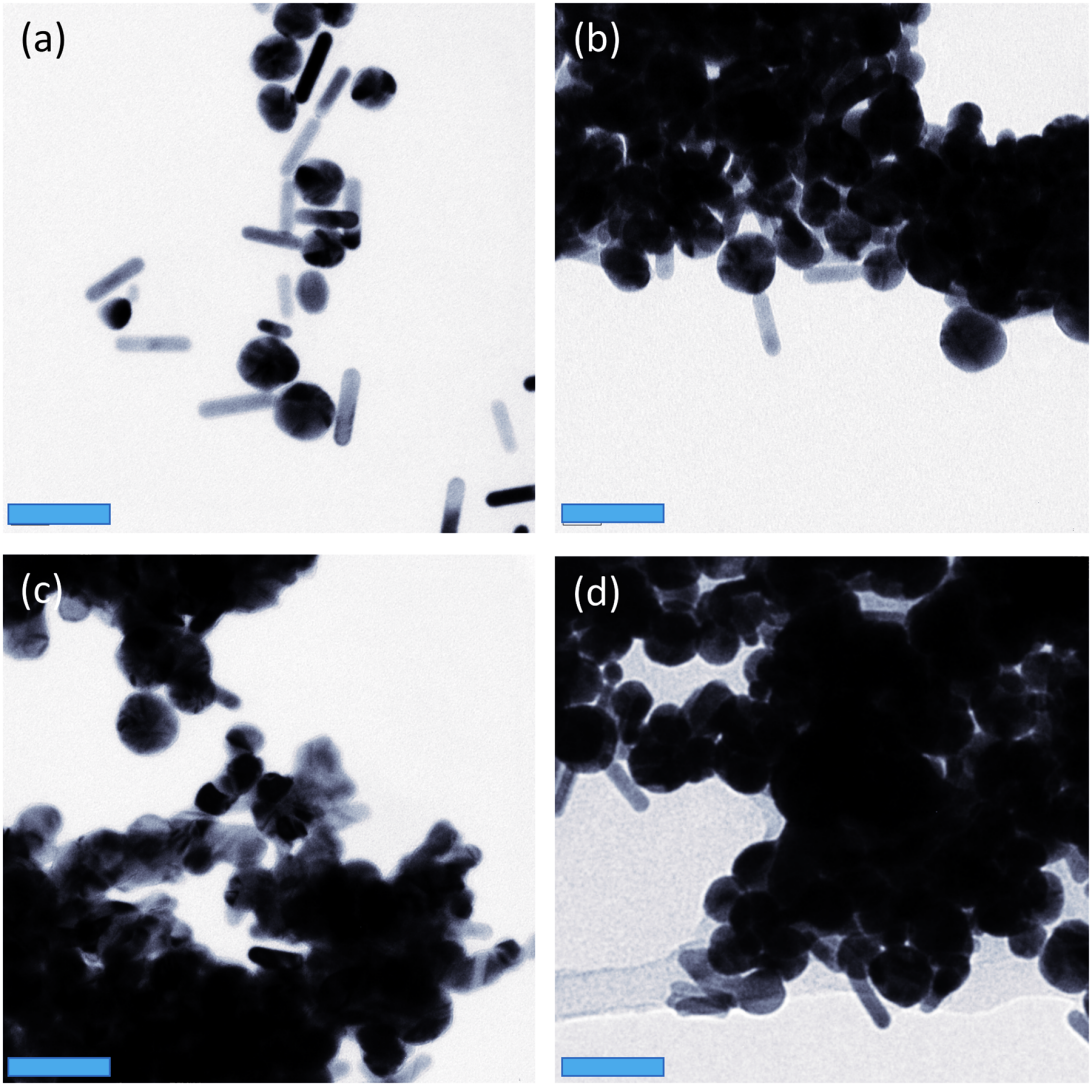
Aggregation of GNRs can be observed in these TEM images (a) representing gold nanorod sample before CW laser irradiation and (b-d) after CW laser irradiation (60,000x magnification). The blue scale bars are 50 nm in length.

**Fig 7:**
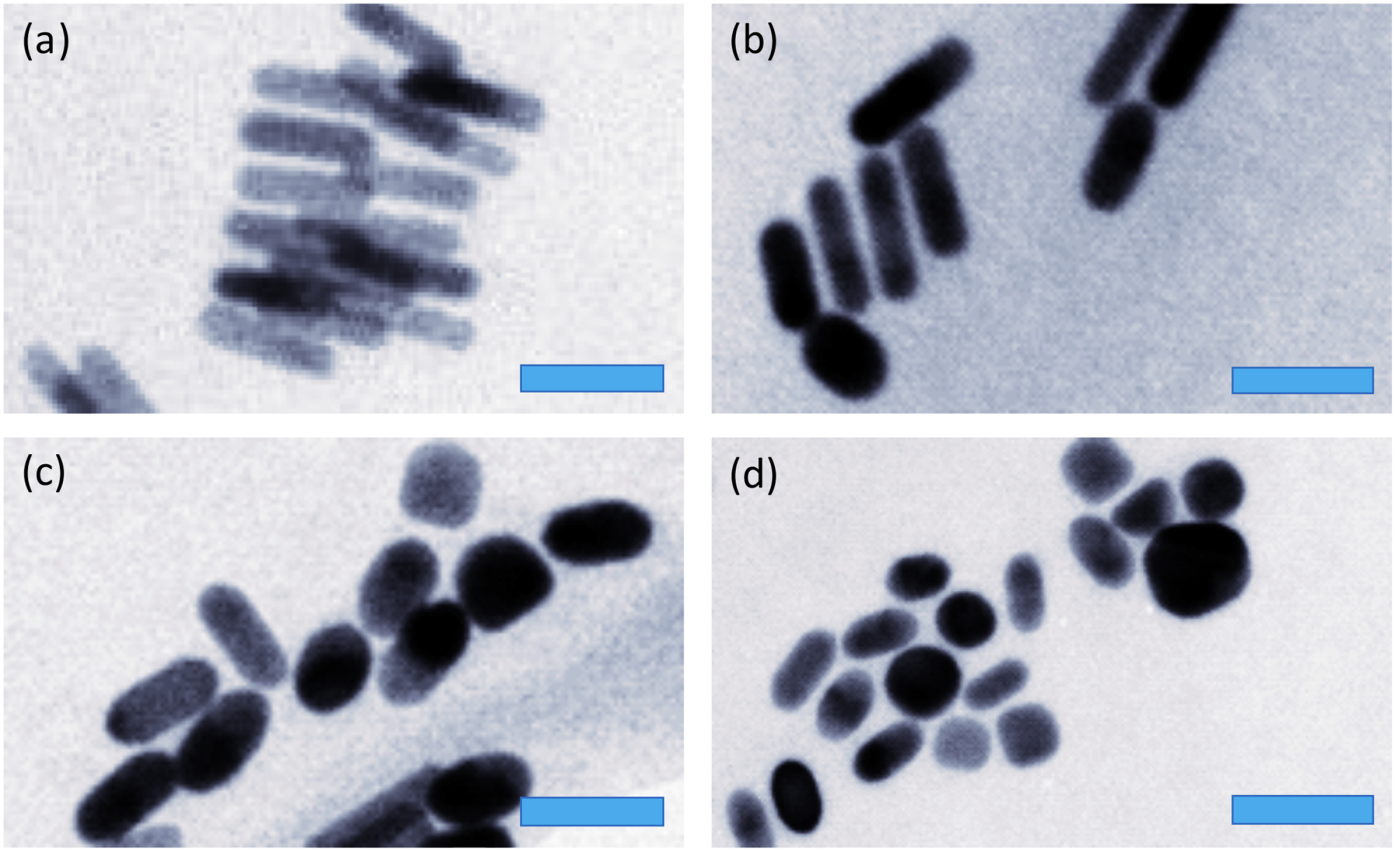
Representative TEM images from sample *α* showing GNRs (a) before laser irradiation, (b) after only PA pulsed laser light, (c) after 6 W/cm^2^ CW irradiation, (d) after 12 W/cm^2^ CW irradiation. The blue scale bars are 50 nm in length.

TEM images from the preliminary GNR sample with the higher GNR concentration that was irradiated is shown in Fig 6 (a–pre-irradiation and b, c, d–post-irradiation), and provided an early indication that reshaping and aggregation may be occurring to this irradiated sample. All subsequent study samples showed corresponding changes in nanorod shape after CW irradiation, confirming that the spectral changes seen were in fact due to shape changes; an example comparison is shown in Fig 7. It appears that the nanorods become shorter and wider, becoming more oval-shaped after laser heating, even tending towards spheres. When applying only the pulsed PA laser, the nanorods did not show a marked shape change as seen in Fig 6(b).

**Fig 8:**
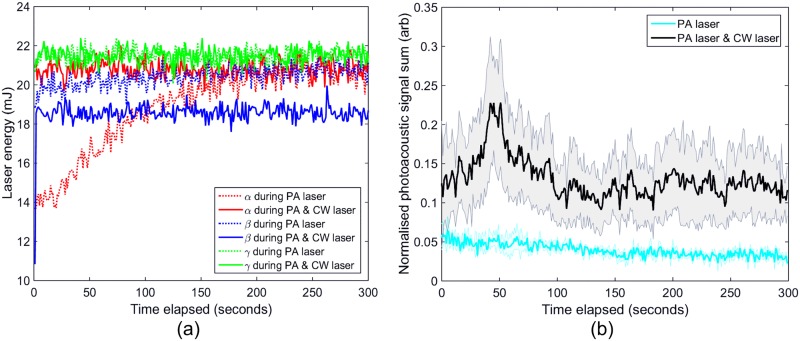
(a) PA pulsed laser energy over time for each acquisition, illustrating the drift and pulse-to-pulse fluctuation in the laser energy, (b) the calculated PA signal mean of the three samples (thick line), with a standard error (shaded area) of the corrected signal, for CW laser on (black) and off (light blue).

The integrated PA signal (the sum of the absolute signal voltage over each interval) is from the ‘top’ and ‘bottom’ of the sample where the signal was low pass filtered using a 15 MHz cut-off and normalised to the laser energy. The means of the samples with and without the CW laser heating are shown in Fig 8(b), with the standard error as the shaded regions and the thick lines as the means. For reference, the PA pulsed laser energy over time for each acquisition is shown in Fig 8(a), illustrating a fluctuation in laser energy. These data were used to remove the laser energy pulse-to-pulse fluctuation from the PA signals by dividing the integrated signals by the energy for each pulse, according to Eq (1).

**Fig 9:**
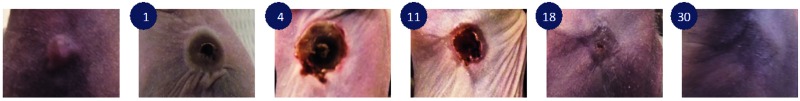
Thermal reaction, gradual healing and complete ablation of tumour xenograft seen *in vivo* in a mouse that received IT GNRs and CW NIR laser irradiation for three minutes. The numbering indicates the days post-irradiation while the first (un-numbered) image is the pre-irradiated tumour. The irradiated site was excised at day 30 and TEM analysis is shown in Fig 10(d)–10(h).

Mice with tumours that received intratumoural (IT) administration of GNRs and irradiation with CW laser demonstrated a photothermal reaction within the subcutaneous tissues which was clearly observed during the first fortnight (as shown in Fig 9). This corresponded to a specific area of tumour necrosis occuring by virtue of the injected GNRs absorbing NIR light and thence eliciting a tumour photothermal response. Within the third week this organised into a scab which subsequently faded to reveal an underlying healed and tumour-free region by day 30 (Fig 9). Microscopic examination of irradiated tumour sites displayed complete tumour ablation with the complete absence of any proliferating cancer cells. Neo-epithelialsation of epidermal and dermal skin layers was seen to occur through the process of neo-collagenesis and this was confirmed by polarised light microscopy.

**Fig 10:**
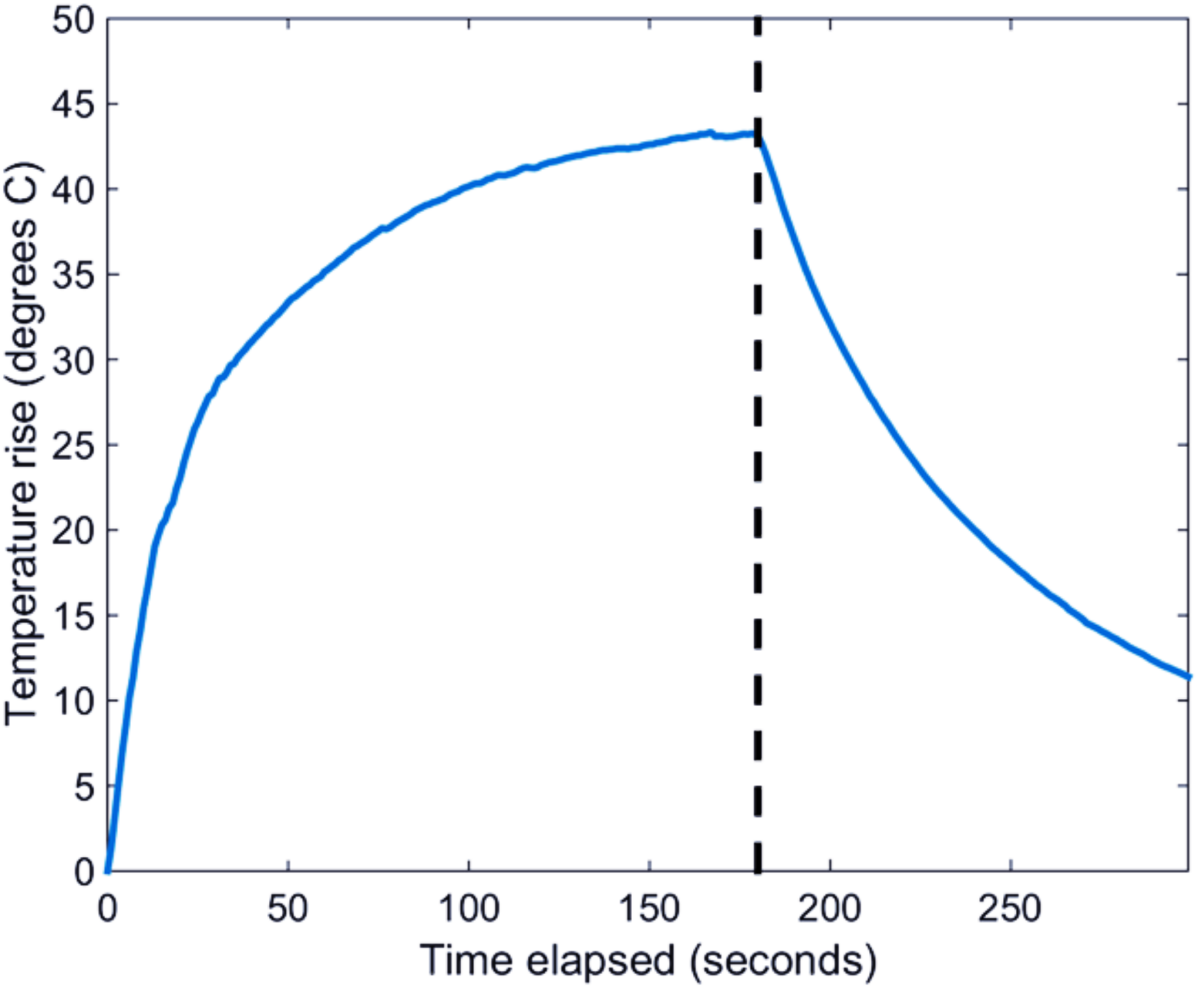
Temperature rise seen *in vivo* after intratumoural (IT) GNRs administration and during CW irradiation for three minutes measured from the tumour site externally by the thermal imaging camera, after adjusting for background and baseline temperatures. The black dashed line shows the time (180 seconds) when the laser was turned off, corresponding to when the ROI reached a 42° temperature rise.

During the three minute period of irradiation with NIR light, the temperature overlying the tumour sites was recorded externally by a thermal imaging camera, and the analysis of the temperature change from baseline is shown in Fig 10. A gradual rise in temperature was observed during irradiation with a final rise of 42°C.

A mean temperature rise of 7.6°C was seen in the CW laser only (without GNRs) control tumours and is most likely arising from skin surface or hair follicle heating, without generating homogeneous heating throughout the tumour. The dearth of any clinical improvement or collateral damage on the irradiated tumour and skin surface illustrated the rather benign nature of NIR irradiation in the absence of GNRs.

**Fig 11:**
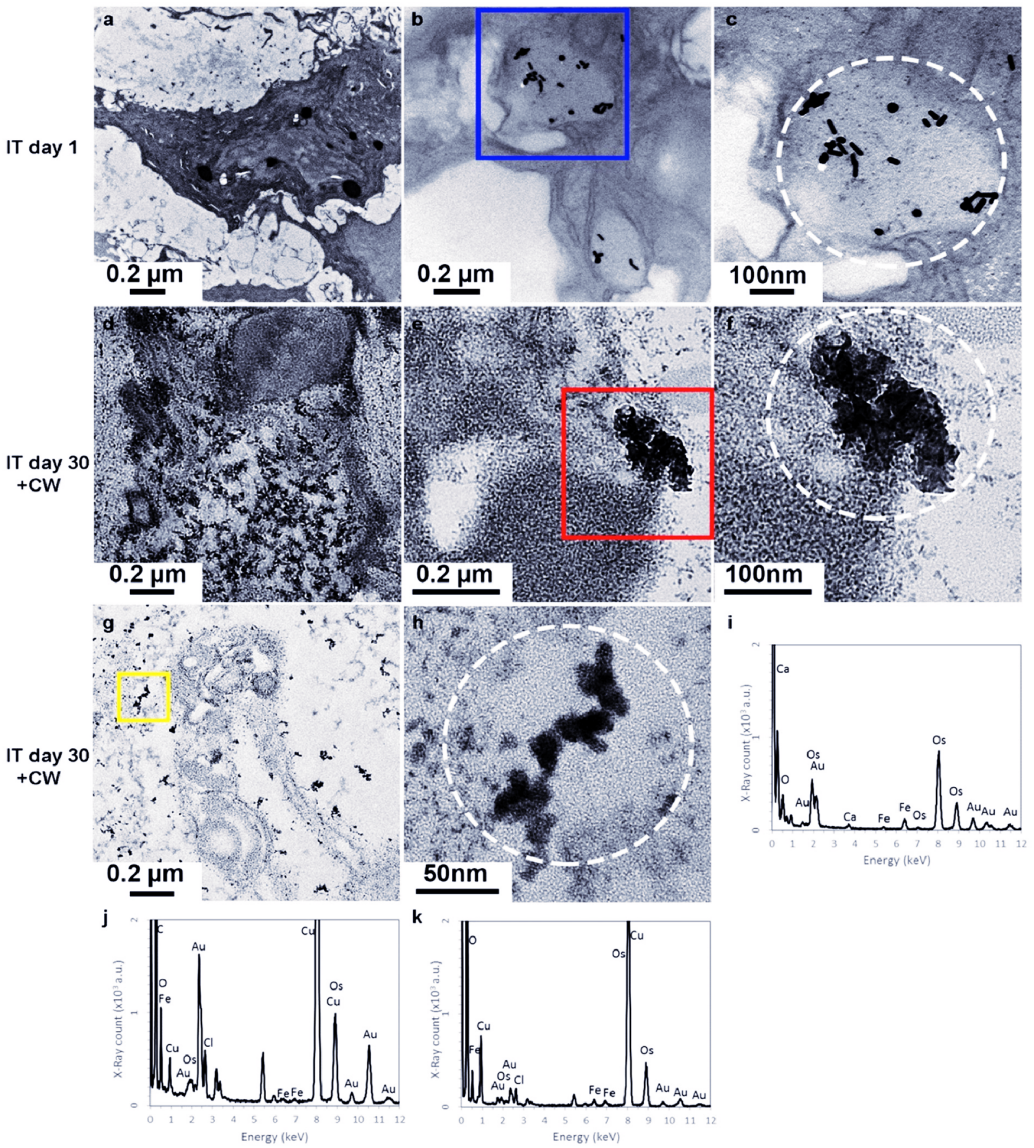
TEM images of in vivo tumour sites following intratumoural (IT) administration of GNRs with pre- (a-c) and post-irradiation (d-h) with CW laser. (a) Low magnification image of day 1 pre-irradiated tumour tissue. (b) GNRs (black rod shaped) were contained in an intracellular (endolysosome) vesicle within a cancer cell. (c) High magnification image of the blue square in (b) showing the preservation of rod-like morphology of endocytosed GNRs pre-irradiation, which were confirmed by Au peaks from TEM-EDX analysis in (i). (d) Low magnification image of the post-irradiated site illustrating the distribution of irradiated particles within the cytoplasm of a single (radiation-damaged) cell. (e) GNR aggregation and reshaping seen within an irradiated cell following CW laser irradiation. (f) High magnification image of the red square in (e). Confirmation that this irregularly shaped structure was in fact morphed GNRs post-irradiation is shown by the presence of Au peaks seen from TEM-EDX analysis (in j). (g) Low magnification image of the post-irradiated site at day 30, illustrating a wide area of reshaped GNRs. (h) High magnification image of the yellow square in (g). The white circles in (c), (f) and (h) correspond to areas analysed and demonstrated as TEM-EDX spectrums in (i), (j) and (k) respectively.

Tumour sites were examined with TEM following intratumoural (IT) administration of GNRs both pre- and post-irradiation with CW laser (Fig 11). These sites were cross-examined with microscopic evaluation to confirm or refute the presence of tumour cells. It was observed that after intratumoural administration, GNRs became endocytosed and were contained within intracellular vesicles within tumour cells as shown in Fig 11(a)–11(c). These pre-irradiated GNRs retained their rod-like morphology and were confirmed to be GNRs by TEM-EDX analysis which corresponded to peaks of Au (gold) precisely at the location of the intracellular rods seen [Fig 11(i)]. TEM images from post-irradiated tumour sites illustrated the biodistribution of GNRs within the cytoplasm of a single (damaged) cell as shown in Fig 11(d). Both GNR aggregation and reshaping were seen within irradiated cells [Fig 11(f) & 11(h)] and these morphed particles were proven to be irradiated GNRs by the presence of Au peaks from TEM-EDX analysis seen in Fig 11(j) & 11(k).

## Discussion

Cytotoxicity of GNPs has been researched in a number of studies. A recent systemic review highlighted some objective evidence into some of the current controversies surrounding their application and their toxicity [31]. The incorporation of a surface coating (such as PEG) has increased the biocompatibility and decreased the cytotoxicity of GNPs. Longitudinal survival studies on mice given varying volumes and concentrations of GNPs demonstrated reassuring objective confirmation that all the animals remained healthy during the study period, with evidence of prolonged survival in other studies [31]. Although there appears to be an initial transient accumulation of gold chiefly in the liver and spleen after intravenous administration, this gradually dissipates sufficiently with no long-term sequelae or signs of toxicity in all *in vivo* studies [31].

The difference in the temperature rise between the samples as seen in Fig 5, can be explained by the difference in their optical absorption at the CW laser wavelength, a phenomenon which agrees with the findings of Chou *et al*. [22]. This shows that the phenomenon of thermally-induced GNR reshaping can occur with a bulk temperature rise as low as 10-20°C, well below the bulk melting point of gold as described by Taylor *et al*. [32]. This however was a global temperature measurement and the local temperature of the centre of the heating laser spot would be much higher and the local temperature at the rod heating centre even higher. Due to convection currents, the GNRs will likely move in and out of the laser spot allowing the majority of the sample to be irradiated.

The heat profiles also showed that there was no heating in the sample due to the PA pulsed laser although some exhibited a slight decrease in temperature. This small decrease is both due to cooling of the sample by the water bath giving an actual temperature decrease and the background ROI showing an apparent warming over time, due to temperature instability of the system, causing an apparent decrease in temperature rise after background subtraction. When the CW laser was applied to the sample, an increase in PA signal up to about 50 seconds was detected, as shown in Fig 8(b). This is most probably due to the heating of the sample which is known to increase the Grueneisen coefficient and therefore increase the effective PA signal, as shown by Shah *et al*. [33]. The Grueneisen parameter (Γ) is defined as the factor which converts absorbed light energy into acoustic pressure:

$$p_{0}=\mu_{a}F_{0}\Gamma ,$$

where *μ*_*a*_ is the optical absorption coefficient and *F*_0_ and *p*_0_ are the fluence and pressure at the sample [33]. The temperature is the only factor which changes the Grueneisen parameter in this type of experiment as the Grueneisen parameter is also defined as $\Gamma =\frac{\beta c^{2}}{C_{p}}$, where *c* is the sound speed, *β* is the thermal expansion coefficient, and *C*_*p*_ is the specific heat capacity of the sample, of which *c* and *β* are temperature dependent [33].

After 50–60 seconds there was a gradual decrease in the PA signal, even though the temperature remained high. This suggests that the optical absorption coefficient of the sample was decreasing, since by this time point the Grueneisen coefficient should have stabilised, and this is confirmed by the absorbance spectra in Fig 4 which clearly show a decrease. A correction factor could potentially be applied using knowledge of water and GNR Grueneisen temperature dependence, enabling probing of the actual change in absorption coefficient, although this could only be performed once the Grueneisen parameter of this type of solution as a function of temperature is known, also to make the results quantitative the system specific variables such as transducer sensitivity would need to be factored in. Observation of the PA absorption spectrum changes continuously during PTT, to observe when the GNRs change their shape, would indeed be interesting and may be a direction for future work. The results would depend on many PTT exposure and tissue variables.

This study demonstrates for the first time that GNRs reshape both *in vitro* and *in vivo* after CW irradiation and that reshaping is a sustained and irreversible phenomenon. These changes were sustained throughout and beyond the period of irradiation because a blue-shift and a considerable diminution in the absorption peak of GNRs had transpired, thus rendering any subsequent CW irradiation at the previous wavelength largely ineffective. Doubling the laser fluence (done here by reducing the laser beam diameter) led to a more dramatic change in morphology and absorption spectra. From the TEM images, it was shown that reshaping occurs in GNRs within five minutes of applying a CW laser *in vitro* and three minutes *in vivo*. This was demonstrated *in vivo* by applying NIR irradiation to the tumour at a fluence comparable to that used for PTT with CW lasers. This was in concordance with a systematic review of the irradiation regimes used by other studies for *in vivo* photothermal therapy purposes [31]. Cancer cells showed that pre-irradiated functionalised GNRs delivered intratumourally appear to become endocytosed, contained within endolysosomes of tumour cells and retain their original shape as demonstrated in Fig 11(b) and 11(c). Proof that these were cancer cells was obtained by examining one half of the biopsied tumour tissue with haematoxylin and eosin staining under light microscopy which showed that the specimen comprised well established adenocarcinoma from an alimentary tract origin.

Confirmation that the particles seen in TEM images were in fact GNRs was obtained by EDX analysis [Fig 11(i) and 11(j)], which corresponded to peaks of Au and other organic compounds intrinsic to cells (C, O and Ca), those used as fixatives (Os and Fe) and the TEM grid (Cu). Post-irradiation aggregation and reshaping was observed at day 30 as spherical GNPs were found within the cytoplasm of membrane-damaged cells as shown in Fig 11(d). Irradiation not only damaged tumour cell membranes but also resulted in the destruction of cellular organelles. It was further evident that these GNPs were found in the cytoplasm and did not become integral to the nucleus. The aggregation and reshaping seen *in vivo* [Fig 11(f) and 11(h)] was indeed comparable to *in vitro* results [Fig 6(b)–6(d)]. The temperature rise seen in the tumour three minutes after CW irradiation was shown in Fig 10, and was greater than the temperature changes seen *in vitro*, likely from the heterogeneous and cumulative heating of cancerous tissue within a living organism. More detailed scrutiny of the reshaped GNRs under high power magnification in Fig 11(h) appears to illustrate a melting of nanorods. Histological assessment of the excised irradiated site demonstrated an absence of proliferating cancerous cells, with regeneration of new epithelium through the process of neo-collagenesis. Hence microscopic evaluation of irradiated sites was congruous with the clinical tumour regression seen macroscopically in Fig 9.

This result is important because CW lasers are the most commonly applied mode of laser irradiation on gold particles for photothermal therapy and it is noteworthy that any subsequent application of laser at the original wavelength is unlikely to produce cumulative or enhanced thermal effects due to the spectral shift and attenuation described here. As heat intensity increased, the blue-shift increased and the aspect ratio reduced. The GNRs also demonstrated a morphological change from rods to spherical-like particles, as shown in Table 1 and in TEM images. For example, sample α changes from an original aspect ratio of 3.8 to 2.9 after CW laser heating, decreasing further to 2.3 when the laser fluence is increased. Ng and Cheng [34] also described this phenomenon by subjecting GNRs to various annealing temperatures using water baths; they found that LSPR bands decay exponentially with annealing time at all annealing temperatures.

While previous studies have shown that GNRs reshape using highly energetic pulsed nanosecond or femtosecond laser light pulse [11–19], a comparison of reshaping with CW lasers and lasers as a function of pulse duration (and duty cycle), down to femtosecond pulse lengths, would be an exciting future study. This would require using multiple lasers with various pulse lengths and various duty cycles, repeating the irradiations, spectral measurements and electron microscopy measurements which might make the experiment quite expensive. This paper focuses on the optical and thermal properties of nanoparticles and of their size, showing that GNRs can change shape using a continuous wave laser irradiation. It has been shown by Gordel et al. that using a femotosecond laser irradiation on GNRs can create nanoparticles of a banana shape [15], however in this study with CW laser irradiation most reshaped GNRs were observed to be shorter and wider ellipse-shapes, as shown in Fig 7 and Table 1.

When comparing the GNR absorbance spectra and temperature rise due to PA and CW lasers, it is apparent that the CW laser caused a much greater heating and spectral hole-burning than the PA laser, as demonstrated in Figs 4 and 5. The average power using the PA laser over 1 second is 20.3 mW with a peak power of 2.9 MW, compared to the 6 W constant power of the CW laser. Therefore much more energy was deposited over the period of the illumination from the CW laser compared to the PA laser, leading to more GNR reshaping. It is clear that in comparing these two regimes, the determining factor in reshaping GNRs through laser irradiation is the average power from the laser rather than its peak power, although this may not be the case for different laser pulse properties. Heat diffusion will also affect heating, and consequently reshaping, as there would be less heat lost if same energy was applied over a shorter time period.

The results from the spectra shown in Fig 4 and the TEM analysis shown in Table 1 suggest that both the lengths of the GNRs and the number of GNRs are reduced after CW laser heating. The original lengths and aspect ratios of GNRs, which resulted in the absorption peak, have reduced, accompanied by a corresponding increase in their widths, as shown in Table 1, and the spectral blue shift after CW laser heating in Fig 4. Furthermore the total number of GNRs may have reduced, as Fig 4 shows the irradiated GNRs LSPR peak is not just blue shifted, the area under the LSPR peak is also reduced and has caused an increase in the TSPR peak at around 530nm, suggesting a conversion from rods to spheres. Prolonging the irradiation period may make all rods ultimately transform into spheres.

Although CTAB-GNRs were used in our *in vitro* studies, it has been previously shown that surface coating with PEG (thiol-terminated polyethylene glycol) also presented a similar attenuation in LSPR absorption coefficients following irradiation with a nanosecond pulsed-laser, with the authors concluding that polymers such as PEG are not an effective heat barrier against the thermally-driven reshaping phenomenon [35]. This concept was then proven with the reshaping of PEGylated GNRs seen in our *in vivo* study. CTAB-GNRs and mutifunctionalised PEG-GNRs are analogous in that they share identical absorption spectra, thus their specific heat capacity would also be the same. Hence utilising CTAB-GNRs or PEGylated GNRs for *in vivo* tissues would generate identical heating results as tissues have constant optical properties. As it is well established that CTAB-GNRs are cytotoxic, thus functionalised and fully coated (PEGylated) GNRs were adopted for biocompatibility purposes for *in vivo* experiments.

Significant aggregation was also seen as a separate entity alongside thermal reshaping post CW irradiation at fluences comparable to photothermal therapy, which would further affect the subsequent optical absorption capacity of GNRs [as seen *in vitro* in Fig 6(b)–6(d) and *in vivo* in Fig 11(f)]. The TEM images of Figs 6(b)–6(d), 7(c), 7(d) and 11(d)–11(h) were all taken after the irradiated GNRs had cooled to room temperature, indicating an irreversible element to the aggregation. A hypothesis that may expound this is that the CTAB coating melted and dissociated from the surface of GNRs, eliminating the buffer interface and hence reducing the inter-particle distances. This has been studied in copolymer conjugation of GNPs *via in situ* small-angle X-ray scattering by Hamner and Maye [36]. A further theory into the causation of this effect has been proposed by Heo *et al*. [37] where the authors suggest that at temperatures beyond 150°C achieved by annealing, aggregation occurs due to multiple hydrogen bonds being broken between random copolymer ligands of nanocomposites. Nam *et al*. [38] suggest that aggregates form rapidly *in vitro* due to electrostatic attractions between “smart” nanoparticles (pH-responsive and without any antibody conjugation), and a gradual accumulation occurs within cells as their exocytosis (clearance) from cells is blocked by the large size of the amorphous complexes. Aggregation was clinically demonstrated to be useful, selective and effective in cancer cells being irradiated with a 660 nm CW diode laser delivered at a relatively low fluence [38]. In a biological environment it is possible that aggregation within cells and reshaping within warm acid environments may modify the optical properties of the tissue, further complicating prediction of the thermal response of irradiating GNR within tissue. It has been shown that GNRs can undergo spectral blue-shifting when in the presence of butenoic acid at 60 degrees Celsius, changing shape from rods, to ellipses, to octahedral [39]. It may be possible that the aggregation effect is reduced by using silica-coated GNRs as they may maintain their surface coating and thus reduce the ability for the nanorods to stick together after heating [40, 41].

The spectral hole burning and shape change illustrated while using a CW laser with fluences comparable to those used in photothermal therapy are comparable to those demonstrated in the literature for pulsed laser irradiation. Therefore the effect of GNR reshaping during CW laser photothermal heating is a clearly observable occurrence, and should be taken into account when planning clinical tumour treatment using this method. Moreover photoacoustic measurements of GNR reshaping due to CW irradiation is demonstrated here, showing potential as a monitoring tool.

## Conclusions

To our knowledge, this is the first time reshaping has been shown using a CW laser both *in vitro* and in successful tumour ablations *in vivo*, which forms the most commonly applied method of laser irradiation on gold nanoparticles for photothermal therapy. It is therefore recommended that the evolution of nanorod morphology be considered during *in vivo* thermal therapy studies in conjunction with light fluence to better appreciate the implications on tumour response, especially where sequential photothermal therapy is being contemplated for therapeutic tumour regression. Reshaping of GNRs after the initial laser application will render subsequent and prolonged irradiations largely ineffective and unlikely to provide any additional clinical effect due to rapid and irreversible alterations in their optical absorption properties.
